# Gas–Water Two-Phase Flow Mechanisms in Deep Tight Gas Reservoirs: Insights from Nanofluidics

**DOI:** 10.3390/nano15201601

**Published:** 2025-10-21

**Authors:** Xuehao Pei, Li Dai, Cuili Wang, Junjie Zhong, Xingnan Ren, Zengding Wang, Chaofu Peng, Qihui Zhang, Ningtao Zhang

**Affiliations:** 1 Tarim Oilfield Company, PetroChina, Korla 841000, China; 2 R&D Center for Ultra-Deep Complex Reservoir Exploration and Development, China National Petroleum Corporation (CNPC), Korla 841000, China; 3 Xinjiang Key Laboratory of Ultra-Deep Oil and Gas, Korla 841000, China; 4 Key Laboratory of Gas Reservoir Formation and Development, China National Petroleum Corporation (CNPC), Korla 841000, China; 5 State Key Laboratory of Deep Oil and Gas, China University of Petroleum (East China), Qingdao 266580, China

**Keywords:** deep tight gas reservoirs, nanofluidics, multiscale porous media, capillary imbibition, threshold pressure gradient

## Abstract

Understanding gas–water two-phase flow mechanisms in deep tight gas reservoirs is critical for improving production performance and mitigating water invasion. However, the effects of pore-throat-fracture multiscale structures on gas–water flow remain inadequately understood, particularly under high-temperature and high-pressure conditions (HT/HP). In this study, we developed visualizable multiscale throat-pore and throat-pore-fracture physical nanofluidic chip models (feature sizes 500 nm–100 μm) parameterized with Keshen block geological data in the Tarim Basin. We then established an HT/HP nanofluidic platform (rated to 240 °C, 120 MPa; operated at 100 °C, 100 MPa) and, using optical microscopy, directly visualized spontaneous water imbibition and gas–water displacement in the throat-pore and throat-pore-fracture nanofluidic chips and quantified fluid saturation, front velocity, and threshold pressure gradients. The results revealed that the spontaneous imbibition process follows a three-stage evolution controlled by capillarity, gas compression, and pore-scale heterogeneity. Nanoscale throats and microscale pores exhibit good connectivity, facilitating rapid imbibition without significant scale-induced resistance. In contrast, 100 μm fractures create preferential flow paths, leading to enhanced micro-scale water locking and faster gas–water equilibrium. The matrix gas displacement threshold gradient remains below 0.3 MPa/cm, with the cross-scale Jamin effect—rather than capillarity—dominating displacement resistance. At higher pressure gradients (~1 MPa/cm), water is efficiently expelled to low saturations via nanoscale throat networks. This work provides an experimental platform for visualizing gas–water flow in multiscale porous media under ultra-high temperature and pressure conditions and offers mechanistic insights to guide gas injection strategies and water management in deep tight gas reservoirs.

## 1. Introduction

Natural gas has emerged as a critical energy source in the global transition toward a low-carbon future [1,2]. Owing to its high combustion efficiency and significantly lower emissions of carbon dioxide, sulfur oxides, and particulate matter compared with coal and oil, it is widely regarded as a clean and efficient bridge fuel [3,4]. As countries strive to achieve carbon neutrality and diversify their energy portfolios, natural gas plays a vital role in balancing environmental sustainability and energy security [5,6]. In particular, the increasing global demand—driven by industrialization, urbanization, and decarbonization policies—has accelerated the shift from conventional shallow reservoirs to deep tight reservoirs [7,8], placing renewed emphasis on technological innovation for efficient natural gas recovery [9,10]. In China, the Tarim Basin hosts one of the richest deep tight gas resources [11], particularly within the Kuqa Depression [12]. As a strategic focus of the Tarim Oilfield, large-scale gas fields such as Kela-2, Dina-2, Dabei, Keshen, and Bozi have been successfully developed in this region [13]. These deep, tight sandstone reservoirs are typically buried at depths of 5500–8100 m, subjected to high temperatures (106–175 °C) and ultra-high pressures (88.9–150 MPa). Their matrix properties are extremely poor, with an average porosity of 5.2% and permeability of only 0.07 mD. In addition, the reservoirs exhibit intense multiscale natural and hydraulic fractures. These complex and extreme geological conditions give rise to intricate gas–water flow behavior [13,14], characterized by high flow resistance [15,16], early water invasion [15,17], and significant water-blocking effects [18,19]. The rise of water is primarily driven by capillary forces in the nanoscale pore network, which facilitate water migration into initially gas-filled pores. Meanwhile, the appearance of gas-liquid two-phase flow results from the interplay between pressure gradients, heterogeneous pore-throat structures, and differences in fluid viscosity and wettability, leading to simultaneous movement of both phases within the porous medium. These issues often result in a short water-free gas production period and a rapid decline in individual well productivity, thereby posing major obstacles to achieving stable output and enhanced gas recovery [20]. As a result, a deeper understanding of the gas–water two-phase flow mechanisms in deep tight formations is essential for optimizing development strategies and improving long-term productivity [21].

Laboratory experiments play a pivotal role in elucidating pore-scale gas–water displacement mechanisms in nanoporous media, while also serving as a critical foundation for validating simulations, refining theoretical models, and informing field development strategies. Core experiments have provided valuable insights into gas–water two-phase flow behaviors at the pore scale. For example, Tian et al. [22] studied the effect of water saturation on the threshold pressure gradient in tight formation. They found that gas threshold pressure gradient (TPG), which is the minimum pressure gradient required to initiate gas flow in porous media, exists at connate water saturation and increases exponentially with either an increase in dimensionless water saturation or a decrease in permeability. Liu et al. [23] analyzed extensive experimental data on gas-driven water displacement and dynamic water invasion and developed methods to quantify dynamic water saturation and capillary pressure. Core experiments offer realistic pore structures but often suffer from poor reproducibility and a lack of direct pore-scale visualization, which limits our understanding of microscale fluid transport mechanisms [24].

Nanofluidics is the study and manipulation of fluids at the nanoscale, typically in channels with dimensions ranging from 1 to 100 nm, where surface and molecular interactions significantly influence fluid behavior [24]. This emerging experimental approach integrates semiconductor microfabrication techniques to investigate fluid heat and mass transfer behavior within nanoporous media [24,25]. It offers several advantages, including nanometer-scale fabrication precision, optical visualization, short testing cycles, and high reproducibility [25,26]. Nanofluidics has been widely applied in various fields, including hydrocarbon production, membrane separation, geothermal extraction, groundwater and soil pollution remediation, geological carbon sequestration, and nano-lubrication [25]. It has recently been applied in the field of unconventional oil and gas recovery [27,28]. For example, Wang et al. [29] developed, for the first time, a nanofluidic slim-tube method to directly visualize the miscible behavior of CO_2_-hydrocarbon systems within multiscale porous media featuring pore sizes from 100 nm to 10 μm. Zhong et al. [11], inspired by the actual porosity and permeability of shale core samples, developed a two-dimensionally confined nanofluidic network (nanoscale depth and width) and achieved quantitative characterization of gas-oil displacement at the 10^1^ nm scale. However, nanofluidic studies focusing on gas–water two-phase flow in multiscale nanoporous media remain extremely limited. Therefore, further investigation is needed to elucidate the pore-scale flow characteristics of gas–water systems in such complex porous structures.

To address this gap, multiscale throat-pore and throat-pore-fracture models based on reservoir data from the Keshen block in the Tarim Basin were designed and fabricated, accurately replicating the complex heterogeneity of natural reservoir systems. A nanofluidic experimental platform capable of operating under ultra-high temperature and pressure conditions (up to 240 °C and 120 MPa) was further developed. Using this platform, real-time visualization of gas–water flow behavior in multiscale porous media ranging from 500 nm to 100 μm under in-situ reservoir conditions (100 °C and 100 MPa) was achieved. The multiphase flow mechanisms in tight reservoirs—including spontaneous water imbibition, threshold pressure gradients, and gas-driven water displacement behavior in multiscale porous media—were systematically revealed. This study establishes a robust platform for visualizing multiscale gas–water interactions and provides actionable insights for improving gas production and water management in unconventional reservoirs.

## 2. Materials and Method

### 2.1. Nanofluidic Chip Design and Fabrication

**Figure 1:**
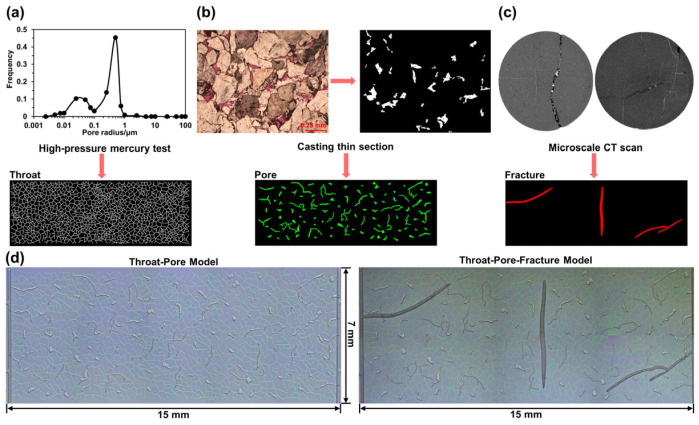
Nanofluidic chip and fabrication. High-pressure mercury test (**a**), casting thin section (**b**), and microscale CT scans (**c**) of Keshen 31 block in the Tarim Basin. (**d**) Multiscale porous media images captured by optical microscopy.

Deep tight gas reservoirs are typically characterized by ultra-low porosity and permeability, complex multiscale throat-pore-fracture systems, and pronounced heterogeneity [30]. This intricate structure gives rise to highly discontinuous and scale-dependent gas–water flow behaviors, posing challenges for direct observation and analysis using conventional experimental methods. To investigate the gas–water flow behavior under multiscale structural coupling, two representative nanofluidic chip models were designed based on the geological characteristics of the Keshen 31 block in the Tarim Basin (Figure 1).

The first model, referred to as the throat-pore model, features a porous medium composed of microscale pores hydraulically connected by nanoscale throats, designed to simulate the tight matrix region of the Keshen 31 reservoir. In this model, pores are spatially isolated and connected solely through narrow constrictions, forming a typical throat-pore network that reflects the dominant transport characteristics of the reservoir matrix, where gas and water must traverse tortuous, high-resistance pathways governed by capillary effects and geometric constraints. According to high-pressure mercury intrusion tests (Figure 1a), pore radii in the Keshen 31 block are predominantly centered at ~250 nm, while cast thin sections (Figure 1b) indicate that intergranular pore radii are mainly distributed around 15 μm. Based on these data, the nanofluidic chip was designed with etch depths of 500 nm (throats) and 30 μm (pores). The morphology and connectivity of the throat network were generated using a Voronoi-based algorithm, yielding a randomly distributed network. Pore geometries were directly extracted from thin-section image analysis to ensure that the model faithfully replicates the natural pore architecture of the target formation.

The second model, referred to as the throat-pore-fracture model, represents the coupling between the tight matrix and larger-scale fractures commonly observed in fractured deep tight gas reservoirs. It was developed by extending the throat-pore model with three microscale fractures, enabling investigation of how fractures influence gas–water flow behavior within multiscale porous media. The morphology and dimensions of the fractures were determined based on micro-CT scans (Figure 1c) of representative reservoir samples. Based on extensive CT image analysis, fracture geometries were classified into two typical types: single fractures and conjugate fracture systems. These patterns were directly incorporated into the chip design and etched to a depth of 100 μm, allowing precise replication of the natural fracture features. In both models, the porous-medium region was uniformly set to 15 mm × 7 mm (Figure 1d).

The fabrication of the nanofluidic chip involved a multi-step microfabrication process. First, the designed structural patterns were transferred onto a silicon substrate via photolithography, followed by reactive ion etching to precisely define the throat, pore, and fracture features across scales. To enhance fluid behavior visualization, the etched silicon surface was then coated with a silicon nitride layer of variable thickness, which provides distinct optical contrasts under microscopy [31]. Finally, anodic bonding was employed to permanently seal the structured silicon wafer to a borosilicate glass substrate, providing excellent mechanical integrity and tolerance to elevated temperatures and pressures.

### 2.2. Experimental Apparatus and Procedure

**Figure 2:**
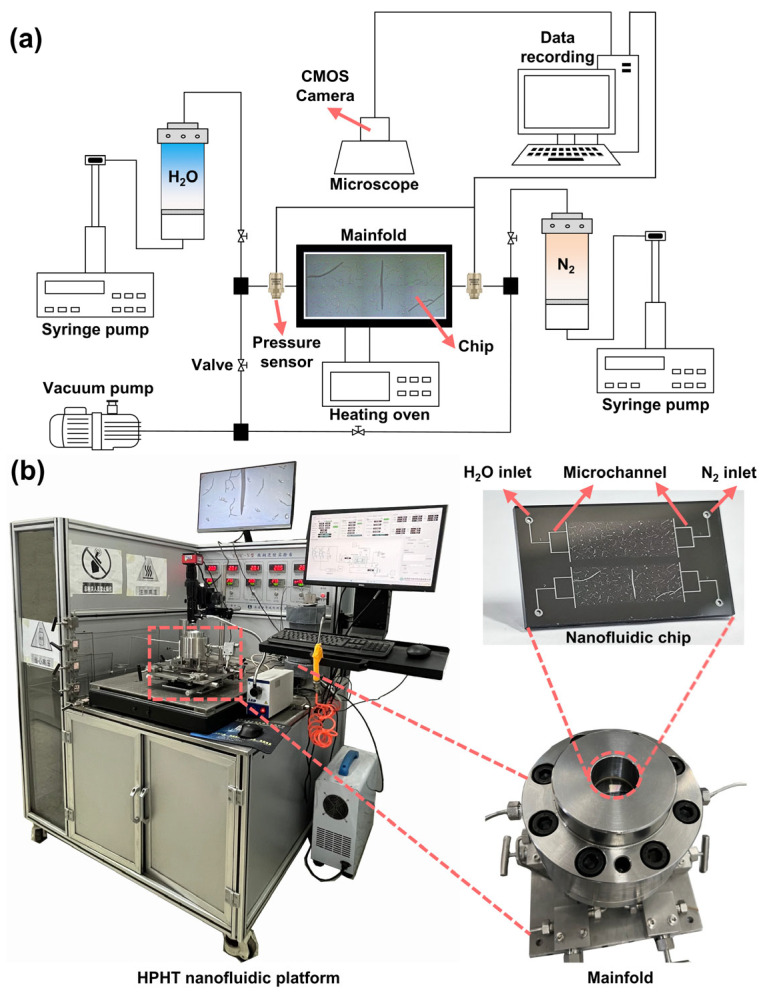
Nanofluidic experiment equipment. (**a**) Schematic of the experimental setup. (**b**) High-temperature and high-pressure (HT/HP) nanofluidic platform.

Figure 2a illustrates the schematic of the experimental setup for studying gas–water flow behavior. In each experiment, the nanofluidic chip is embedded within a customized chip manifold sealed with O-rings to ensure pressure integrity during fluid injection. To enhance pressure tolerance, a liquid confining-pressure method is employed. A cavity formed between the chip and the inner surface of the manifold serves as the confining chamber, into which pressurized deionized water is injected to apply uniform external pressure around the chip. The entire manifold is wrapped with a heating mantle, which is controlled by a heating oven to electrically heat both the manifold and the internal fluid. The heating mantle is capable of withstanding temperatures up to 240 °C. A temperature sensor is inserted approximately 1 cm below the chip to monitor temperature. Owing to the short thermal conduction path and the excellent thermal conductivity of both the silicon and the stainless steel, the measured temperature is considered representative of the actual fluid temperature within the chip. Fluid delivery is precisely controlled using high-pressure syringe pumps (XHC-D; pressure resolution: 0.01 MPa), with a maximum operating pressure of 120 MPa, ensuring stable injection under high-pressure conditions. To enable real-time monitoring of injection pressure, two pressure sensors (0.01 MPa resolution) are installed near the nitrogen (N_2_) and water inlets to continuously record fluid pressures. Flow behavior is visualized in real time using a CMOS camera (3840 × 2160 pixels) mounted on an optical microscope (A0-HK830RT). The camera operates at a maximum frame rate of approximately 100 frames per second, enabling the capture of the rapid fluid displacement process. In addition, to ensure the fluid front remains within the camera’s field of view during such fast flows, we have incorporated an electric *XY*-axis moving platform beneath the chip manifold. This platform enables real-time adjustment of the chip holder’s position, ensuring continuous monitoring of the advancing fluid front. To address the challenge of balancing mechanical strength with optical access in high-pressure experiments, we selected a long focal length objective lens with a working distance of 8.6 cm. This choice allowed us to achieve the necessary high magnification while maintaining the structural integrity of the chip holder under high-pressure conditions. The long working distance minimizes optical distortion and ensures high-resolution imaging, enabling us to capture detailed images despite the mechanical constraints imposed by the pressure requirements. Additionally, the design of the chip holder was optimized to minimize its thickness, ensuring sufficient optical access while maintaining the required strength for high-pressure operation. All components described above are integrated into a compact high-temperature, high-pressure nanofluidic platform capable of withstanding temperatures up to 240 °C and pressures up to 120 MPa, enabling robust experimental performance under extreme operating conditions (Figure 2b).

Prior to fluid injection, the entire system is evacuated with a vacuum pump to eliminate residual air. The manifold and chip are then rapidly heated to 100 °C (reservoir temperature) using the heating mantle. The experiments consist of two sequential stages: a water-imbibition test followed by a gas-injection test for water-lock removal. The gas-injection stage is conducted after establishing a two-phase gas–water distribution through spontaneous water imbibition. For safety under high-temperature and high-pressure conditions, N_2_ is used as a substitute for natural gas. This substitution is justified by the fact that N_2_ shares similar transport and phase-behavior characteristics with natural gas in porous media, making it a reasonable proxy.

For the water imbibition experiment, high-purity N_2_ (99.99%) is injected at low pressure through the chip’s N_2_ inlet using a syringe pump connected to an N_2_-filled piston cylinder. The injection pressure is progressively increased until it reaches 100 MPa and is then maintained as a back pressure during the imbibition process, with a control accuracy of 0.01 MPa. Once the N_2_ pressure stabilizes at 100 MPa, deionized H_2_O is injected through the chip’s H_2_O inlet using another syringe pump connected to a piston cylinder. The water is initially introduced at low pressure and gradually pressurized to 101 MPa. When the N_2_-water interface approaches the microchannels near the water inlet, the water injection pressure is promptly adjusted from 101 MPa to 100 MPa to eliminate further forced flow. As the interface advances into the porous media, water imbibition becomes entirely driven by capillary forces. During this stage, real-time imaging is conducted through both photographs and video recordings to monitor fluid motion. Once the N_2_-water distribution stabilizes and phase saturations no longer change with time, the imbibition experiment is considered complete. Subsequently, N_2_ injection is initiated to investigate water-lock removal. The water pressure is kept constant at 100 MPa, while the N_2_ pressure is gradually increased in increments of 0.01 MPa. At each pressure step, a 10-min hold is applied to observe movement of the N_2_-water interface. The pressure difference at which the interface begins to move within a specific scale of porous medium is defined as the threshold pressure for that medium. Once all scales of the porous structure exhibit interface movement, gas injection continues under various constant pressure differentials. The resulting gas–water flow behavior and phase distributions are continuously observed and recorded.

### 2.3. Image Processing and Analysis

**Figure 3:**
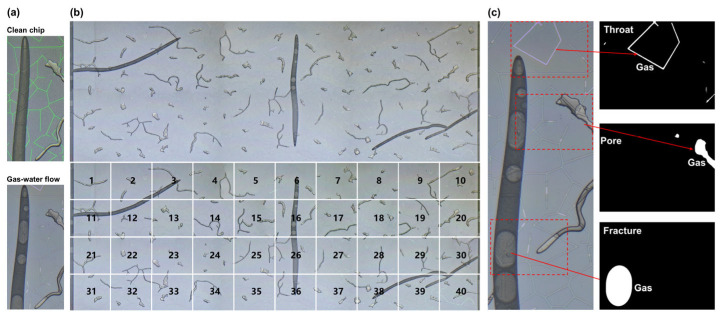
Methods of image processing. (**a**) Captured representative images at different stages. (**b**) Division of the nanofluidic chip into 40 subregions for scale-resolved spatial analysis. (**c**) Fluid phase segmentation in multiscale porous media based on color thresholding.

To better distinguish and illustrate different fluids in porous media, we show two representative captured images at different stages (Figure 3a). In the clean nanofluidic chip, the colors reflected by the porous media at different scales vary. This variation is attributed to the presence of a silicon nitride thin film coating on the channel surface, as well as differences in light reflection caused by the varying depths of the channels. The colors observed in the throats, pores, and fractures are green, white, and grey, respectively, reflecting the unique interactions of light with the media at different depths and structural features. In the nanofluidic chip saturated with water, we observed that the water appeared dark gray in the fractures and pores, while it appeared light gray in the throats. When high-pressure gas is introduced into the porous media, the high-pressure gas appeared bright white in the fractures and pores and pale pink in the throats. These images clearly demonstrate the shift in color, reflecting the transition between different fluid phases in the chip, and the corresponding changes in the color distribution within the pores.

During each experiment, we captured the entire nanofluidic chip by taking images, which were then stitched together to form a large composite image. This image was subsequently divided into 40 subregions for fluid phase saturation analysis (Figure 3b). Fluid phase saturation is precisely quantified by processing captured image sequences with a color threshold algorithm in ImageJ v1.54p software. The RGB color space serves as the identification threshold, enabling the distinction of N_2_, H_2_O, and porous media (Figure 3c), and allowing accurate identification of the N_2_ phase (white regions) at each pore size scale [29]. For each scale, Equation (1) is used to calculate the N_2_ saturation (*R*) as follows:

(1) $$R=\frac{A_{N_{2}}}{A_{\mathrm{porous}}}\times 100\%$$

where $A_{porous}$ is the area of the pore space at the specific scale; $A_{N_{2}}$ is the area of the N_2_ phase within that pore space.

## 3. Results and Discussion

### 3.1. Water Imbibition Dynamics in Throat-Pore Structures

**Figure 4:**
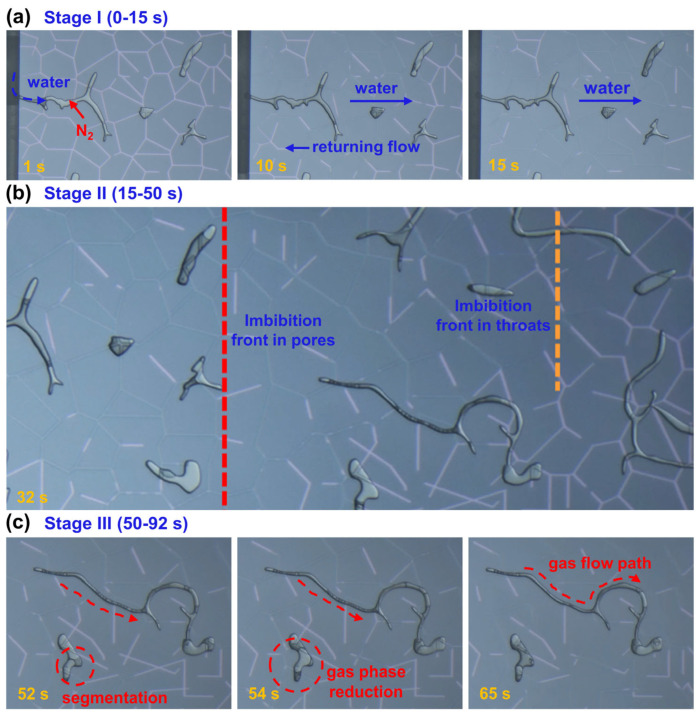
Visualization of spontaneous water imbibition in the throat-pore model at 100 °C and 100 MPa. The process is divided into Stage I (**a**), Stage II (**b**), and Stage III (**c**).

To investigate the dynamics of spontaneous water imbibition in throat-pore structures, we conducted nanofluidic experiments at 100 °C and 100 MPa using a multiscale throat-pore model. Based on the flow characteristics of the water phase and the evolution of the two-phase distribution, the process can be divided into three stages over 92 s (Figure 4). In Stage I (Figure 4a, 0–15 s), water spontaneously enters the porous media driven by capillary forces. Due to the significantly higher capillary pressure in throats (500 nm) than in the pores (30 μm), water preferentially invades the throat regions, forming continuous water pathways [32]. As the throats become saturated, water gradually advances into the adjacent pores, where larger pore size and lower capillary pressure lead to slower imbibition. This selective invasion results in non-uniform water distribution and localized water backflow, driven by capillary pressure imbalance or reverse gradients caused by gas compression. The velocity contrast between throat and pore flow further intensifies the dynamic heterogeneity at the imbibition front.

As imbibition progresses to Stage II (Figure 4b, 15–50 s), the water front continues to advance rapidly along the connected throat network, while the pores experience delayed filling due to increasing gas compression. The residual gas forms localized blockages, generating counteracting forces that hinder further water invasion at the throat-pore junctions. This marks a transition from a purely capillary-driven process to a capillary-gas coupling regime, further amplifying the contrast between throat and pore dynamics.

In Stage III (Figure 4c, 50–92 s), the trapped gas undergoes gradual mobilization. Continued water invasion compresses and segments the gas into isolated clusters, which are progressively displaced, releasing additional pore space. Local gas breakthrough occurs when the internal gas pressure exceeds the capillary entry pressure, allowing gas to escape through high-permeability channels. These processes lead to dynamic rearrangement of gas–water interfaces, maintaining a highly heterogeneous two-phase distribution despite the overall increase in water saturation. After 92 s, the water front fully breaks through the porous medium, marking the end of the dynamic imbibition front propagation. Beyond this point, the water pathways stabilize, and no further large-scale front advancement is observed. The subsequent evolution is dominated by local redistribution of the two-phase fluids, primarily reflected in gradual saturation adjustments rather than interface displacement. This post-breakthrough period represents a quasi-equilibrium stage, during which capillary-driven redistribution continues until residual saturation levels stabilize. Although the dynamic imbibition process is visualized up to 92 s, fluid saturation is continuously monitored for over 30 min to capture this subsequent quasi-equilibrium behavior.

### 3.2. Water Imbibition Dynamics in Throat-Pore-Fracture Structure

**Figure 5:**
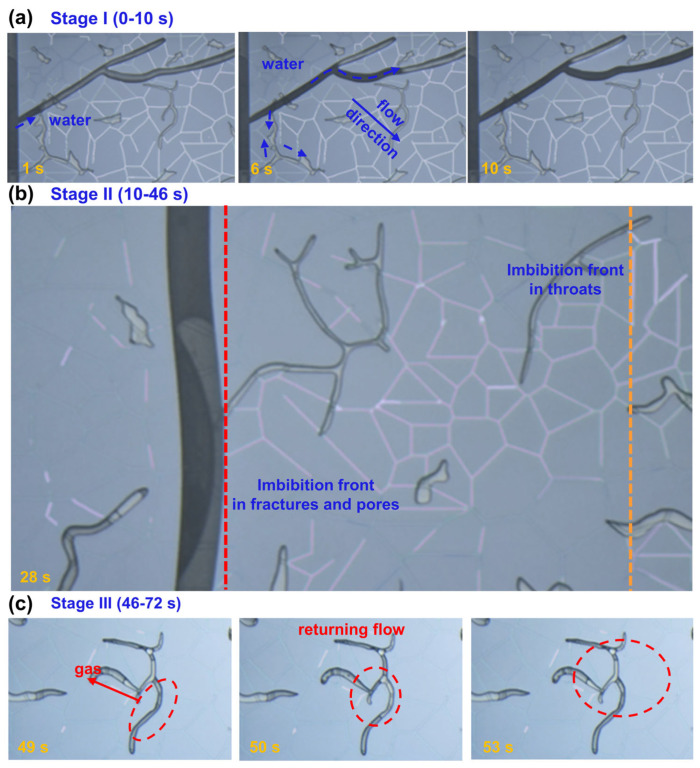
Visualization of spontaneous water imbibition in the throat-pore-fracture model at 100 °C and 100 MPa. The process is divided into Stage I (**a**), Stage II (**b**), and Stage III (**c**).

To investigate the dynamics of spontaneous water imbibition in throat-pore-fracture structures and clarify how fractures influence imbibition behavior in heterogeneous porous media, we conducted nanofluidic experiments at 100 °C and 100 MPa using the throat-pore-fracture model. Similar to the throat-pore model, the throat-pore-fracture model also follows three stages over 72 s (Figure 5). In Stage I of imbibition (Figure 5a, 0–10 s), water spontaneously invades the porous media under capillary forces. Due to the fracture’s high permeability and direct connectivity to the injection channel, water preferentially enters the fracture network, establishing a rapid fluid conduit. The fractures serve as preferential flow paths, allowing the water phase to bypass some of the local throat-pore resistance and quickly reach regions further from the inlet. As the fractures become saturated, water redistributes into adjacent throats and neighboring pores. Although capillary suction still drives water into smaller throats and pores, the fracture’s guidance effect accelerates the overall front propagation and alters the initial imbibition pattern compared with a fracture-free system.

In Stage II of imbibition (Figure 5b, 10–46 s), the water front continues to advance rapidly through the connected throats, while imbibition in isolated pores lags behind. When the front reaches the vertical fractures, propagation dynamics change due to structural heterogeneity. Specifically, variations in fracture aperture lead to non-uniform advancement: the central region of the vertical fracture exhibits slower front movement compared with the edges, resulting in a concave imbibition profile within the fracture plane. Moreover, in areas where the fracture does not penetrate throats, the throat network maintains continuity, allowing the water front to bypass the fracture plane locally. In these zones, the imbibition front in the throats propagates significantly faster than in the neighboring pores, and the contrast between rapid throat flow and delayed pore filling becomes increasingly pronounced after water passes through the vertical fracture.

In Stage III of imbibition (Figure 5c, 46–72 s), the water-swept region begins to mobilize the residual gas trapped in the porous structure. As water continues to invade, the gas is progressively compressed and segmented into isolated clusters, which are gradually displaced, releasing additional pore space. Simultaneously, localized gas breakthrough occurs when the internal gas pressure exceeds capillary barriers, allowing gas to escape through the percolated fracture network or locally high-permeability pathways. These events lead to dynamic rearrangement of the gas–water interfaces, further complicating the two-phase distribution. Compared with the fracture-free system, the fractures accelerate gas release but also introduce additional heterogeneity in the displacement pattern. After 72 s, the water front fully breaks through the throat-pore-fracture system, and the flow pathways stabilize. Further evolution is mainly characterized by gradual saturation redistribution within the pores and throats, while the fractures maintain stable flow conduits. Similar to the throat-pore system, fluid saturation is continuously monitored beyond this point to capture post-breakthrough redistribution and final equilibrium.

Overall, the fractures not only accelerate the initial imbibition but also reshape the multiscale displacement dynamics, introducing additional flow pathways and modifying the gas-liquid interface evolution.

**Figure 6:**
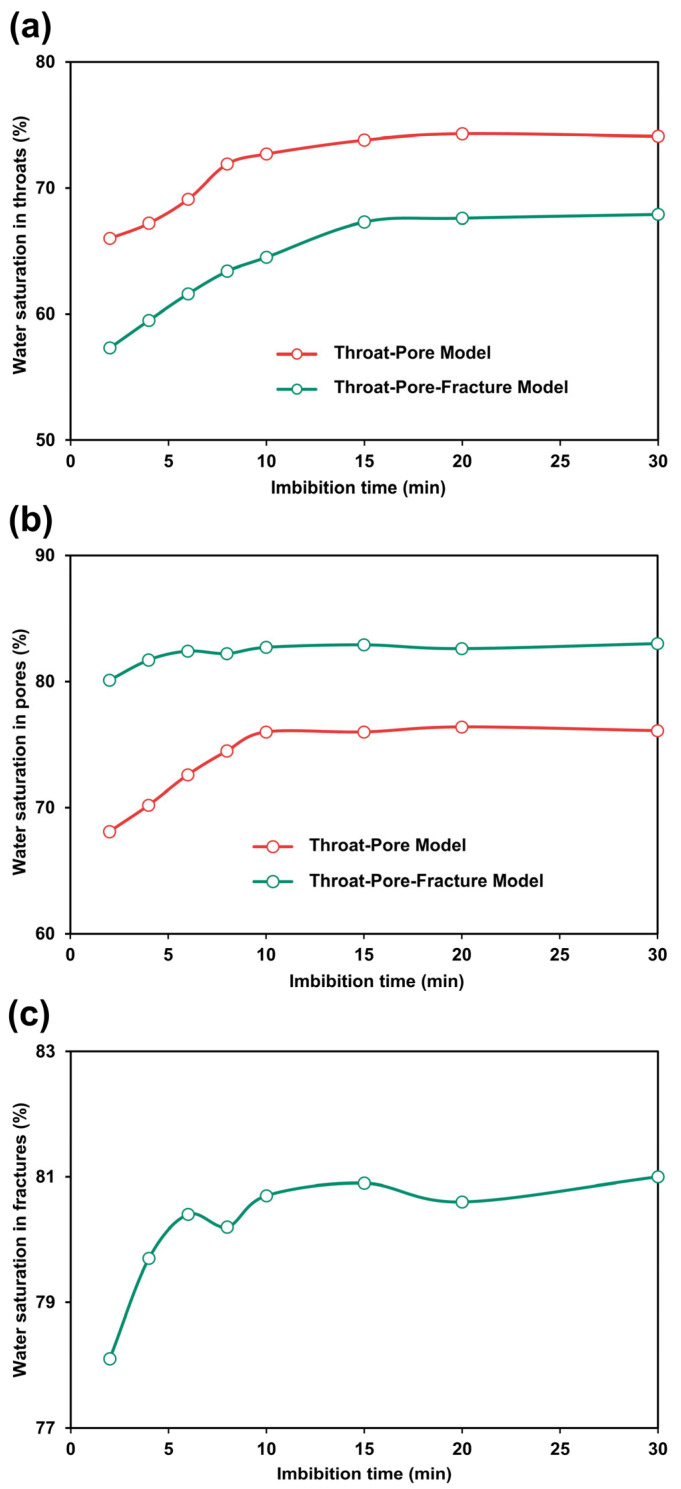
Temporal evolution of water saturation in throats (**a**), pores (**b**) and fractures (**c**), for the throat-pore and throat-pore-fracture models. The imbibition time starts from the moment the water front first enters the porous media.

We further quantified the temporal evolution of water saturation within different pore-scale domains in both the throat-pore and throat-pore-fracture models (Figure 6). In multiscale porous media, capillary pressure is predominantly controlled by nanoscale throats, making their saturation dynamics a key characteristic of the imbibition process. Figure 6a shows the time-dependent evolution of water saturation in 500 nm throats for both models. In the throat-pore model, throat water saturation increases from 66% at 2 min to 73% at 10 min, eventually stabilizing at 74% by 30 min. In contrast, the throat-pore-fracture model exhibits both slower saturation growth and a lower final saturation: throat water saturation rises from 57% at 2 min to 66% at 15 min, reaching only 67% by 30 min. These differences highlight the impact of fractures on multiscale fluid displacement. The throat-pore-fracture model incorporates three additional fractures with widths on the order of 100 μm. During imbibition, the water phase preferentially enters these high-permeability fractures, establishing rapid flow conduits that fundamentally alter the displacement dynamics. The presence of fractures induces two coupled effects: a substantial portion of the invading water bypasses the throats and flows directly toward the outlet, weakening the capillary-driven invasion into the throat network; meanwhile, the rapid flow in the fractures compresses and traps gas within the throats, generating localized gas-blocking phenomena that further suppress water entry. As a result, the throat-pore-fracture model exhibits slower throat filling and a lower final water saturation in throats.

Figure 6b shows the time-dependent evolution of water saturation in 30-μm pores in the throat-pore and throat-pore-fracture models. In the throat-pore model, water saturation in the pores increases from 68.13% at 2 min to 75.86% at 10 min, gradually stabilizing at 76.14% by 30 min. In contrast, the throat-pore-fracture model shows a faster saturation rise in pores, increasing from 57% at 2 min to 82.4% at 6 min, and reaching 83% at 30 min. This difference is attributed to the presence of fractures, which act as high-permeability conduits connecting multiple pores and forming efficient imbibition pathways. By enhancing the overall connectivity of the pore system, the fractures facilitate faster water entry and result in a higher final water saturation in the throat-pore-fracture model compared with the throat-pore model.

Figure 6c shows the time-dependent evolution of water saturation in 100-μm fracture in the throat-pore-fracture models. In the throat-pore-fracture model, the water saturation in fractures increases from 80.02% at 2 min to 82.4% at 6 min and gradually stabilizes at 83% by 30 min. As high-permeability conduits, the fractures are rapidly filled during the early stage of imbibition, making their saturation less sensitive to imbibition time in the later stages.

### 3.3. Threshold Pressure Gradient in Multiscale Porous Media

**Figure 7:**
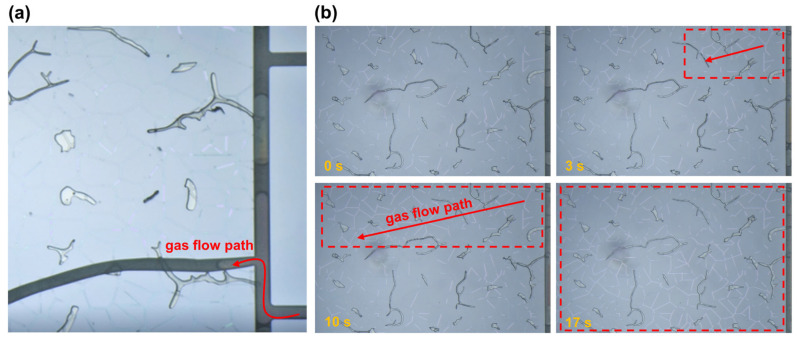
Visualization of gas threshold pressure gradient at 100 °C. (**a**) Gas displacement initiation stage in the throat-pore-fracture model. (**b**) Temporal evolution of gas displacement regions in the throat-pore model.

Understanding the threshold pressure gradient is critical for characterizing capillary-controlled flow behavior in tight and multiscale porous media [33]. In gas–water systems, this threshold defines the minimum pressure required to overcome capillary barriers and initiate gas displacement of water. This phenomenon plays a key role in the production dynamics of tight gas reservoirs, where pore-scale capillary resistance can significantly affect gas deliverability, especially during early-stage depletion or under low-pressure conditions [34]. To investigate this effect, we conducted threshold pressure gradient measurements in both the throat-pore model and the throat-pore-fracture model at 100 °C (Figure 7).

Experimental results show that the threshold pressure gradients of the throat-pore model and the throat-pore-fracture model are relatively close. The measured threshold gradient is 0.25 MPa per centimeter for the throat-pore model and 0.30 MPa per centimeter for the throat-pore-fracture model. During gas-driven water displacement, the gas phase must overcome not only the capillary entry pressure but also additional resistance from interfacial tension fluctuations at the gas–water interface. Consequently, the measured threshold pressure gradients are higher than those predicted from throat capillary entry pressure alone (~0.07 MPa). Capillary forces play a dominant role in controlling the initial gas breakthrough pathways. Due to capillary pressure heterogeneity, the displacement process follows a preferential pathway mechanism, where the largest channels are invaded first. As shown in Figure 7a, gas preferentially displaces water from the fractures during the early stage, then progressively invades the pores and throats. Figure 7b illustrates the dynamic evolution of gas invasion, which exhibits a rapid expansion once mobilization begins. Most of the gas front propagation occurs within the first minute, followed by a gradual stabilization after 15 min, indicating a transition from dynamic displacement to quasi-steady behavior. Notably, the threshold pressure gradients measured in the Keshen-parameterized chips (0.25 and 0.30 MPa cm^−1^ for the throat-pore and throat-pore-fracture models, respectively) are of the same order of magnitude as core-scale values commonly reported for tight sandstones under comparable water saturations and permeabilities [35,36]. This consistency indicates that the large pressure drops observed in tight-reservoir production do not originate solely from pore-throat capillarity; rather, they primarily arise from scale-dependent connectivity bottlenecks and the cross-scale Jamin effect, compounded by sample-scale heterogeneity and fracture-matrix transfer resistance under HT/HP conditions.

### 3.4. Gas Displacement of Water in Multiscale Porous Media

Gas injection is an effective method for mitigating water-blocking and restoring gas flow pathways in tight reservoirs. By displacing water trapped in pore spaces, gas injection re-establishes continuous gas channels, which is critical for enhancing productivity in gas reservoirs, especially during early production stages or following water influx events. A key factor governing the success of gas-driven water displacement is the displacement pressure differential. Selecting an appropriate pressure differential is crucial for balancing efficient water removal with reservoir integrity and operational safety. Insufficient pressure may fail to overcome capillary barriers, leaving water retained in critical flow paths, while excessive pressure can lead to unwanted fracture propagation or gas channeling.

**Figure 8:**
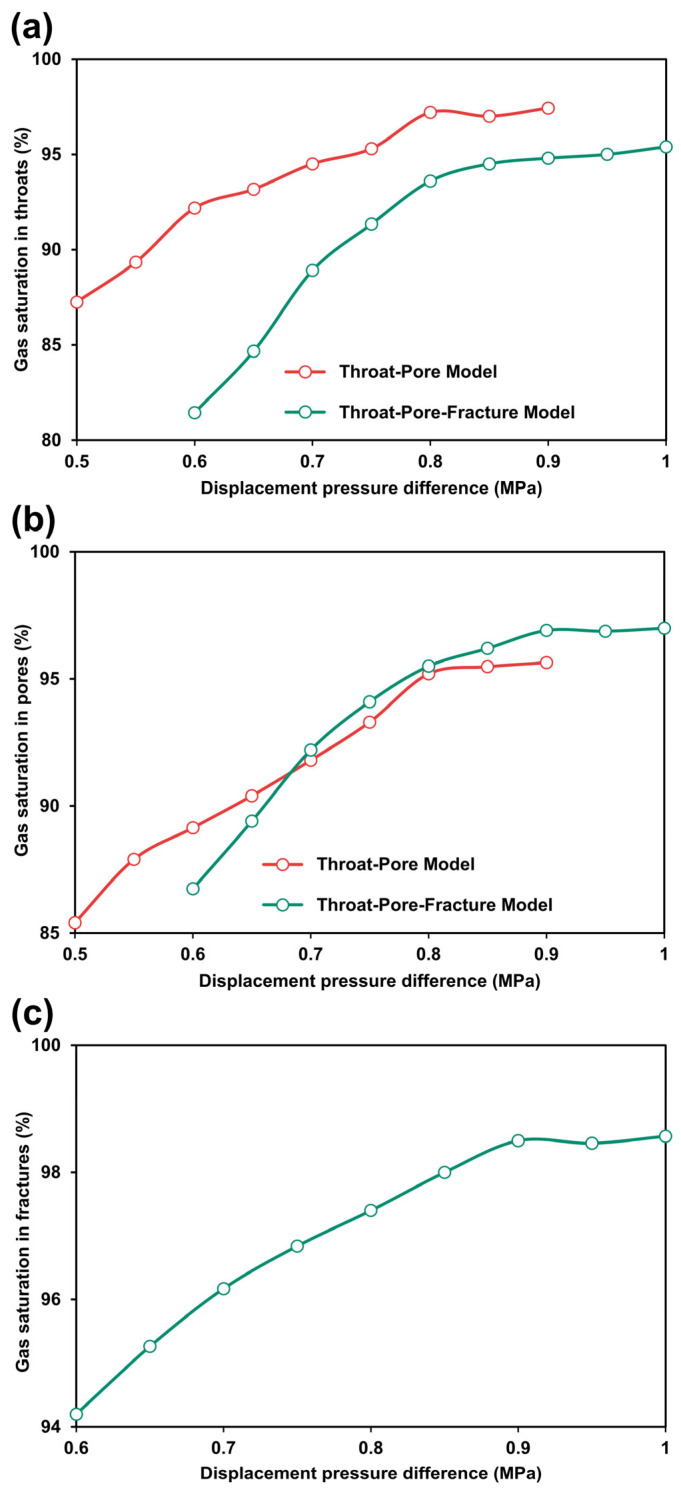
Water saturation response to displacement pressure difference in throats (**a**), pores (**b**) and fractures (**c**), for both the throat-pore and throat-pore-fracture models.

To investigate the effect of the displacement pressure differential on gas displacement efficiency, we conducted a series of gas injection experiments at varying injection pressures in both the throat-pore and throat-pore-fracture models (Figure 8). These experiments were carried out after completing the threshold pressure gradient tests. The evolution of gas saturation in the throats (500 nm) under different displacement pressures is shown in Figure 8a. The throat-pore model exhibits a higher final gas saturation in the throats compared with the throat-pore-fracture model. Specifically, in the throat-pore model, gas saturation increases from 87.25% at 0.5 MPa to 96.2% at 0.9 MPa, resulting in an 8.95% increment. In the throat-pore-fracture model, gas saturation rises from 81.45% at 0.6 MPa to 95.37% at 1.0 MPa, corresponding to a 13.92% increment. This difference indicates that fractures divert a portion of the gas flow, reducing the displacement efficiency in throats and leaving more residual water.

The gas saturation evolution in pores (30 μm) is presented in Figure 8b. In contrast to the throat results, the pores exhibit a higher final gas saturation in the throat-pore-fracture model than in the throat-pore model. In the throat-pore model, gas saturation increases from 85.41% at 0.5 MPa to 95.67% at 0.9 MPa, with a 10.26% change. In the throat-pore-fracture model, it rises from 86.74% at 0.6 MPa to 96.88% at 1.0 MPa, showing a 10.14% change. The higher micropore saturation in the fracture model is attributed to the enhanced connectivity provided by the fractures, which facilitates more effective gas invasion into isolated pores.

For the fractures (100 μm), gas saturation results are shown in Figure 8c. Gas saturation in the fractures increases from 94.2% at 0.6 MPa to 98.57% at 1.0 MPa, corresponding to a relatively small change of 4.37%. Since fractures serve as the primary gas flow conduits, most of the water is displaced at the initial threshold pressure, leading to a higher starting gas saturation compared with pores and throats. Due to their large aperture and lower capillary resistance, the fractures are less sensitive to displacement pressure changes in the later stages.

## 4. Conclusions

In this study, we developed throat-pore and throat-pore-fracture models based on the reservoir characteristics of the Keshen block, accurately replicating the multiscale heterogeneity of tight gas reservoirs. Using these models, we conducted spontaneous imbibition and gas-displacement experiments under in situ reservoir conditions, achieving real-time visualization of gas–water migration in multiscale porous media under high-temperature and high-pressure (HT/HP) conditions.

The results show that in the throat-pore model, nanoscale throats and microscale pores exhibit good connectivity, allowing for rapid spontaneous water imbibition without significant scale-induced resistance. However, the introduction of 100 μm fractures generates preferential flow pathways, leading to enhanced micro-scale water locking effects and faster attainment of gas–water equilibrium. The threshold pressure gradient for gas-driven water displacement in the matrix does not exceed 0.30 MPa/cm, suggesting that capillarity alone (typically below 0.1 MPa) is not the primary controlling factor. Instead, the cross-scale Jamin effect, caused by the combined throat-pore-fracture structure, dominates the displacement resistance. At higher displacement pressure gradients (~1 MPa/cm), water in the matrix can be rapidly expelled to lower saturations due to efficient nanoscale throat connectivity. These findings provide mechanistic insights into multiscale gas–water displacement under realistic reservoir conditions and offer practical guidance for optimizing gas injection operations and improving water management in unconventional gas reservoirs.

Overall, compared with previous studies on gas–water displacement in tight gas reservoirs, our study establishes a micro- and nanoscale cross-scale experimental model operable under HT/HP conditions, a combination that, to our knowledge, has not been systematically investigated in prior experimental work. By directly visualizing and quantifying gas–water migration in multiscale porous media under HT/HP, we provide new insights into displacement mechanisms. These findings are relevant to optimizing gas-injection strategies and improving water management in unconventional gas reservoirs.

## Data Availability

Data are contained within the article.
